# Congenital Central Hypoventilation Syndrome Presenting with Seizures

**DOI:** 10.7759/cureus.6680

**Published:** 2020-01-16

**Authors:** Abdulaziz Binmanee, Abdulrahman Alfadhel, Naif Alzamil, Sara ALBanyan, Mishal Alammar

**Affiliations:** 1 Pediatrics, King Faisal Specialist Hospital and Research Center, Riyadh, SAU; 2 Emergency Medicine, King Faisal Specialist Hospital and Research Center, Riyadh, SAU; 3 Internal Medicine, King Faisal Specialist Hospital and Research Center, Riyadh, SAU; 4 Urology, King Faisal Specialist Hospital and Research Center, Riyadh, SAU

**Keywords:** cchs, phox2b, pulmonology, autonomic nervous system, seizure

## Abstract

Congenital central hypoventilation syndrome (CCHS) is a critical and rare autosomal dominant disorder that was first described by Robert Mellins in 1970. CCHS is defined to be an autonomic nervous system (ANS) dysfunction that usually presents in the neonatal period with hypoventilation and dysregulated autonomic homeostasis on a multi-system level. Classically, CCHS presents with normal ventilation while awake, and hypoventilation with normal respiratory rate during sleep. CCHS has been causally linked to the paired-like homeobox 2B (PHOX2B) gene. We report the case of a full-term male infant diagnosed with CCHS at two months of age with repeated extubation failure secondary to CCHS. The patient was discharged at five months of age with a home ventilator.

## Introduction

Congenital central hypoventilation syndrome (CCHS) is a serious life-threatening autosomal dominant disorder classified as a neurocristopathy; an estimation of CCHS incidence has been reported to be 1 in 200,000 births [1]. Neonates experience apnea and cyanosis during sleep as a consequence of progressive hypoxemia and hypercarbia due to the functional loss of coordinated autonomic regulation of the respiratory system, explained by an attenuated sensory capacity of medullary CO2 receptors [2-5]. Paired-like homeobox 2B (PHOX2B) has been identified to be the disease-defining gene in CCHS and is required to make the diagnosis [4]. The two other manifestations of neurocristopathy, Hirschsprung disease (HSCR), and neuroblastomas (NB) are the commonly associated disorders in CCHS patients as PHOX2B gene mutation increases susceptibility [5].

The severity of CCHS manifestations is highly dependent on the gene mutation variant [6-7]. Severe cases warrant multidisciplinary care and invasive ventilatory assistance, most effectively in the form of positive-pressure ventilation (PPV) along with tracheostomy, while less severe cases can be successfully managed with non-invasive positive-pressure ventilation (NPPV) [5]. We present a case of a full-term male infant diagnosed with CCHS at two months of age with repeated extubation failure, The patient was discharged at five months of age with a home ventilator.

## Case presentation

The patient is a full-term male, product of an uneventful, spontaneous vaginal delivery. Apgar score was nine, five, and six at one, five, and 10 minutes after birth, respectively. He was born to a medically free 33-year-old, gravida 2 para 2 mother, weighing 3780 grams. His older sibling is healthy. Prenatal examinations were unremarkable. Physical examination revealed no abnormality with normal postnatal meconium emission. Six minutes after delivery, and after a vigorous cry, the patient developed respiratory distress and fever, requiring neonatal intensive care unit (NICU) admission.

**Figure 1:**
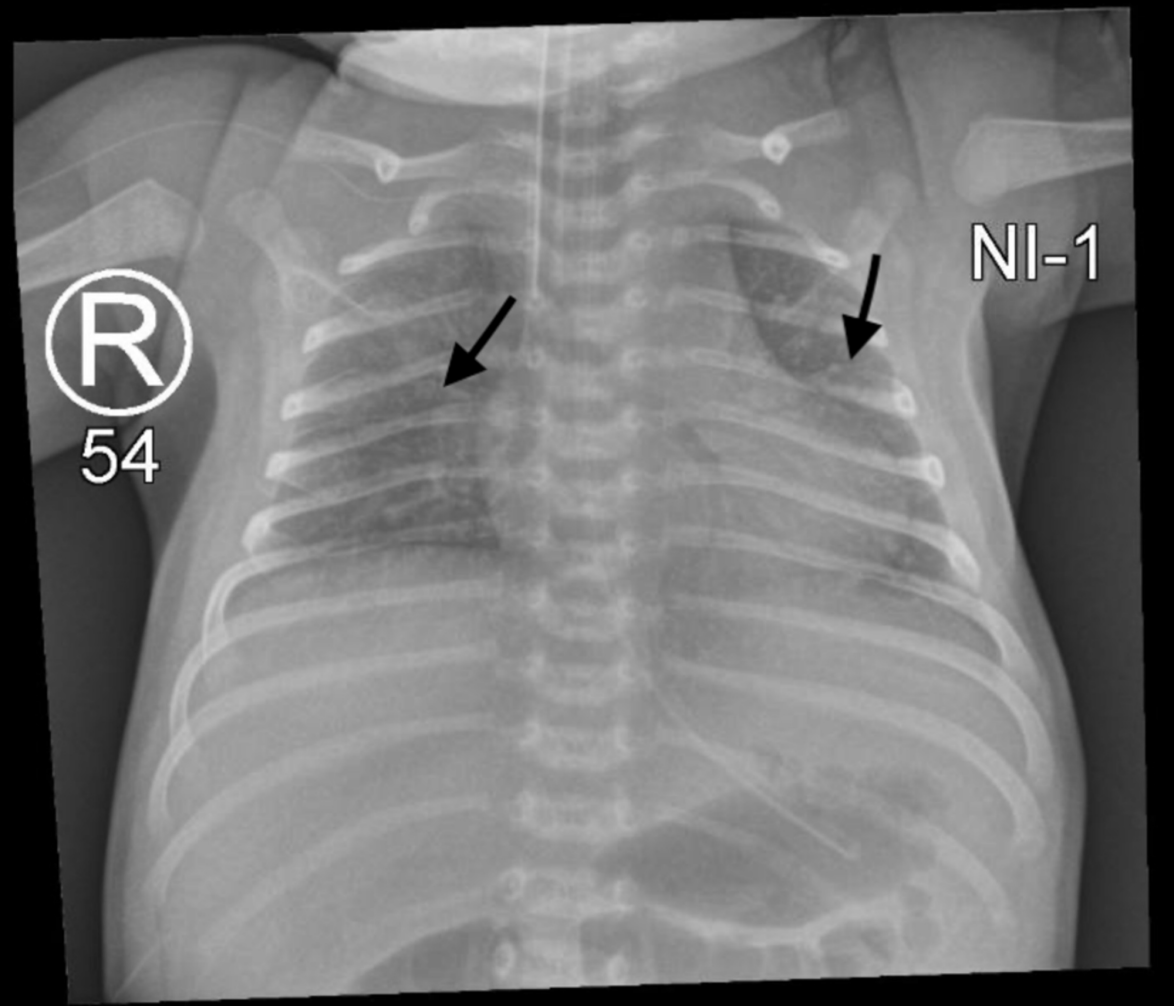
X-ray showing multiple infiltrates

**Figure 2:**
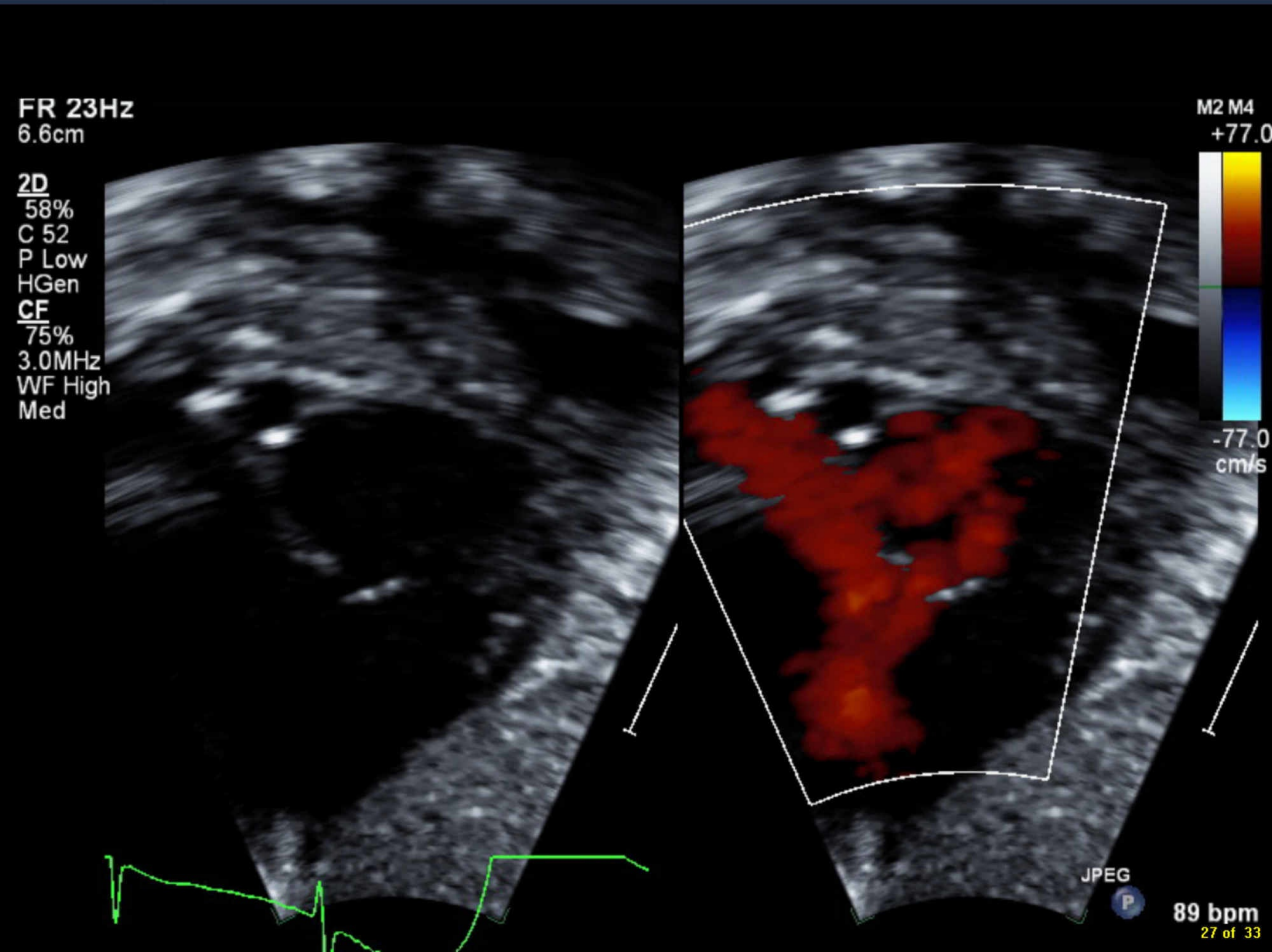
Echocardiography showing patent foramen ovale

Upon NICU admission, empirical antibiotics were initiated. Septic workup came out negative. Chest X-ray exhibited multiple infiltrates involving both lung fields, mainly in the perihilar area (Figure 1). Echocardiography showed patent foramen ovale with a bidirectional shunt, otherwise normal (Figure 2). Cord gases revealed a pH of 6.9 with normal HCO3 levels (22 mEq/L), respiratory in origin, with recurrent apneas and CO2 retention. The patient was thusly intubated. At five days of age, an extubation attempt failed due to frequent apneic episodes associated with lip-smacking and lateralized gazing. 

**Figure 3:**
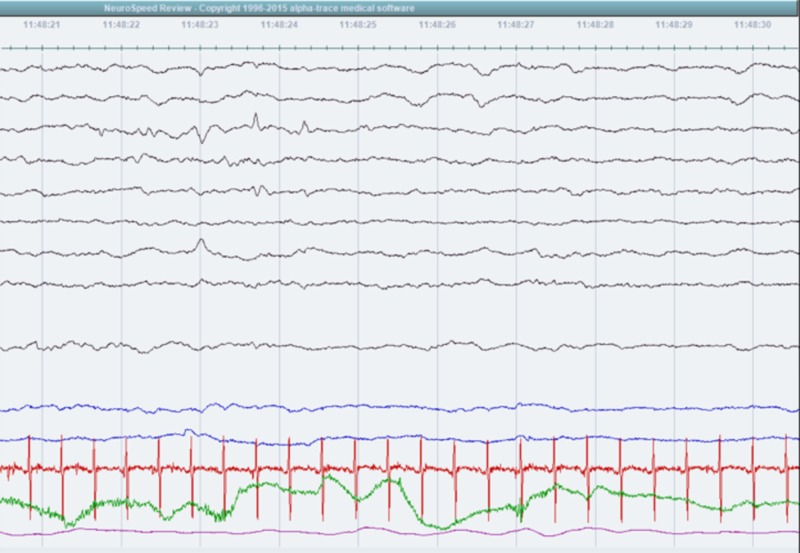
Electroencephalogram showing sharp waves and spikes

Frequent apneic episodes accompanied by seizure features in this patient necessitated comprehensive neurological evaluation to optimize extubation attempts. Lumbar puncture, head ultrasound, and brain magnetic resonance imaging (MRI) were done, showing no abnormal findings. Electroencephalogram (EEG) upon sedation expressed a burst suppression pattern. Follow-up EEGs off-sedation revealed multi-regional, bihemispheric sharp waves and spikes (Figure 3). The patient was started on antiepileptic medications. 

On day six of age, due to recurrent apneas occurring mainly during sleep with body temperature dysregulation, and after two extubation attempts, the patient was suspected to have congenital central hypoventilation syndrome. PHOX2B gene was sent for analysis, revealing a heterozygous polyalanine expansion to 26 repeats located in coding exon 3 of the PHOX2B gene. No variants or non-polyalanine repeats were detected. 

At two weeks of age, the patient tolerated a shift from mechanical to non-invasive ventilation. One week later, he exhibited cyanotic spells, necessitating reversion to mechanical ventilation. A total of seven extubation attempts across three months culminated in failure due to resultant hypoxia and hypercapnia. At two months of age, the patient underwent permanent tracheostomy placement for long-term ventilatory support.

The study subject was discharged at the age of five months, after the optimization of home ventilation settings. Members of the community home health-care program visited him once a month, providing medical support and routine vaccination. His developmental milestones were normal upon the last visit. He remained on a ventilator with tracheostomy during sleep time, and no feeding problems have arisen so far.

## Discussion

CCHS is a genetic autonomic nervous system (ANS) disease that poses a high diagnostic difficulty to clinicians due to its rarity and variable phenotypic nature. Many infants during their neonatal period would lack the classical wakefulness-sleep dyspneic variation [8]. Thus, a high index of suspicion should be maintained throughout the work-up testing. Focused history, physical examination, and comprehensive pulmonary, cardiac and neurological assessments are needed, usually after NICU admission to exclude primary disorders that might explain the clinical manifestations more plausibly in such infants [4]. Patients affected require life-long ventilatory support and follow up visits.

PHOX2B gene, located on chromosome 4p12, encodes a highly conserved paired-like homeobox transcription factor that plays a vital regulatory role in neural crest migration and ANS development. The gene is expressed in central and peripheral ANS during early human embryonic development [9]. The importance of PHOX2B in the pathogenesis of CCHS and other neurocristopathies has been increasingly endorsed since its relevance was first discovered in 2003 [9]. 

CCHS is inherited in an autosomal dominant pattern with incomplete penetrance. However, in most cases, genetic testing of parents reveal no abnormalities, as the majority of reported cases are the result of de novo mutations [4]. More than 90% of CCHS cases revealed in-frame PHOX2B mutations with an expansion of polyalanine repeats in exon 3 [10]. While a heterozygous mutation in PHOX2B is enough to express symptoms of CCHS, the severity of ANS symptoms has been variable within those patients. This has led to the identification of a genotype-phenotype positive correlation in CCHS. The length of polyalanine repeat mutations (PARMs) was shown to be significantly associated with more ANS symptoms as well as longer daily requirements of ventilatory support, however, the number of organs affected did not [9-10]. 

The patient reported here required intubation and mechanical ventilation on the first day of life. In attempting to transition to non-invasive ventilation, multiple extubation attempts were made. The first attempt was followed by cyanosis and seizure-like features which required a comprehensive neurological work-up, in a way to unfold diagnostic uncertainty as well as to optimize extubation attempts. The patient’s EEG suggested non-specific cortical dysfunction that predisposed to focal seizures without secondary generalization. At this stage, the diagnosis of CCHS was highly suspected and genetic testing for PHOX2B mutation was done. The result of the analysis confirmed the diagnosis, revealing a heterozygous polyalanine expansion to 26 repeats located in coding exon 3 of the PHOX2B gene.

After months of in-patient care and comprehensive evaluation and optimization of home ventilation settings, our patient was stable enough to permit home-care and was safely discharged at five months of age with follow-up visits.

## Conclusions

Rapid recognition, respiratory support, and continuous monitoring are the most important steps in the acute management of new CCHS cases. Delaying evaluation and acute care of CCHS leads to significantly lower rates of survival and more morbidities. As in the presented case, most infants will require tracheostomy with PPV to relieve cyanosis and progressive hypercarbia.
